# Microfluidic Impedance Cytometry for Single‐Cell Particulate Inorganic Carbon:Particulate Organic Carbon Measurements of Calcifying Algae

**DOI:** 10.1002/gch2.202200151

**Published:** 2022-12-07

**Authors:** Douwe S. de Bruijn, Dedmer B. Van de Waal, Nico R. Helmsing, Wouter Olthuis, Albert van den Berg

**Affiliations:** ^1^ BIOS Lab‐on‐a‐Chip group MESA+ Institute for Nanotechnology Max Planck—University of Twente Center for Complex Fluid Dynamics University of Twente Drienerlolaan 5 Enschede Overijssel 7522 NB The Netherlands; ^2^ Department of Aquatic Ecology Netherlands Institute of Ecology (NIOO‐KNAW) Droevendaalsesteeg 10 Wageningen 6708 PB The Netherlands

**Keywords:** calcifying algae, microfluidic impedance cytometry, ocean carbon cycle, PIC:POC ratio, single‐cell characterization

## Abstract

Calcifying algae, like coccolithophores, greatly contribute to the oceanic carbon cycle and are therefore of particular interest for ocean carbon models. They play a key role in two processes that are important for the effective CO_2_ flux: The organic carbon pump (photosynthesis) and the inorganic carbon pump (calcification). The relative contribution of calcification and photosynthesis can be measured in algae by the amount of particulate inorganic carbon (PIC) and particulate organic carbon (POC). A microfluidic impedance cytometer is presented, enabling non‐invasive and high‐throughput assessment of the calcification state of single coccolithophore cells. Gradual modification of the exoskeleton by acidification results in a strong linear fit (*R* ^2^ = 0.98) between the average electrical phase and the PIC:POC ratio of the coccolithophore *Emiliania huxleyi* 920/9. The effect of different CO_2_ treatments on the PIC:POC ratio, however, is inconclusive, indicating that there is no strong effect observed for this particular strain. Lower PIC:POC ratios in cultures that grew to higher cell densities are found, which are also recorded with the impedance‐based PIC:POC sensor. The development of this new quantification tool for small volumes paves the way for high‐throughput analysis while applying multi‐variable environmental stressors to support projections of the future marine carbon cycle.

## Graphical abstract

Calcifying algae play an important role in the ocean carbon cycle. The calcification state of these algae is studied at single‐cell level using microfluidic impedance cytometry. This work demonstrates that the algal calcification state can be assessed by the electrical phase. Additionally, the influence of changes in the seawater carbonate chemistry on the calcification state is investigated.

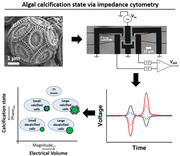

## 1 Introduction

A recent study by the National Academies of Sciences, Engineering, and Medicine published “a research strategy for ocean‐based carbon dioxide removal and sequestration”^[^ ^1^ ^]^ to investigate the potential contribution of oceans to meet the global climate goals of the Paris agreement.^[^ ^2^ ^]^ One of the approaches in this study considers “ocean alkalinity enhancement” to alter the seawater chemistry via alkalinity (e.g., enhanced weathering^[^ ^3^ ^]^) and thereby remove carbon dioxide (CO_2_) from the atmosphere. However, it is unclear how this hypothetical enhancement will impact ocean life, and in particular calcifying organisms.

**Figure 1:**
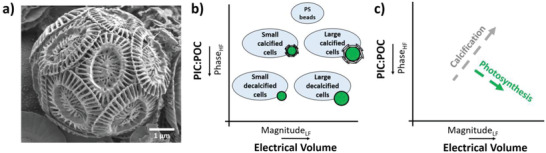
a) SEM image of a single coccolithophore *Emiliania huxleyi*. b) Proposed classification of coccolithophores according to their PIC:POC ratio (via phase) and their electrical volume (via magnitude). The impedance magnitude at low frequency scales proportionally with the volume of a particle and is therefore given as the electrical volume. The phase response of calcified cells is expected to shift toward the phase response of polystyrene (PS) beads, because of their dielectric nature. c) Expected transition as a result of calcification or photosynthesis in coccolithophore cells.

Calcifying algae, like coccolithophores (**Figure** **1**a), greatly contribute to ocean alkalinity and the oceanic carbon cycle in general.^[^ ^4^, ^5^ ^]^ They play a key role in two processes of the marine carbon cycle that both, in turn, are important for the effective CO_2_ flux: The organic carbon pump (photosynthesis) and the inorganic carbon pump (calcification).^[^ ^6^ ^]^ The fixation of CO_2_ via photosynthesis into organic carbon (particulate organic carbon (POC)) results in the drawdown of CO_2_ in the surface ocean, whereas the formation of the calcium carbonate exoskeleton (particulate inorganic carbon (PIC)) results, counter‐intuitively, in a net release of CO_2_ (Ca^2+^ + 2HCO_3_^−^ → CaCO_3_ + H_2_O + CO_2_^[^ ^6^ ^]^). Besides, the formation of the exoskeleton adds ballast to the algae, resulting in a sinking flux of carbon to the deep ocean.^[^ ^7^, ^8^ ^]^ The ratio between the exported inorganic and organic carbon to deeper ocean layers (i.e., the “rain ratio”) is of particular interest for ocean carbon models and the forecast of the future marine carbon cycle.^[^ ^9^, ^10^, ^11^, ^12^, ^13^, ^14^, ^15^ ^]^ The relative contribution of calcification versus photosynthesis in calcifying algae, and thereby the potential for inorganic and organic carbon that can be exported, is expressed by the algal PIC:POC ratio.

Among the major challenges in understanding impacts of climate change on calcifying algae, and their feed‐back to the marine carbon cycle, is the potential for interactive effects across a wide range of environmental variables (e.g., CO_2_ concentration, temperature, nutrients, and irradiation). Lab‐on‐a‐chip technology can greatly help testing the vast number of combinations of environmental variables, owing to its ability to precisely control culture conditions at high‐throughput.^[^ ^16^, ^17^ ^]^ Recent research demonstrates the biological response of microalgae to nanoplastics under 2000+ conditions.^[^ ^18^ ^]^ These systems typically work with small volumes, for which we would require a tool to analyze the PIC:POC ratio for small amounts of cells. Recent efforts show single‐cell measurements using fluorescent labeling of the coccoliths,^[^ ^19^ ^]^ optics and dissolution of the exoskeleton by electrochemistry,^[^ ^20^ ^]^ and differentiation of cells with and without exoskeleton by impedance flow cytometry.^[^ ^21^ ^]^ Up till now, however, these methods have limited sensitivity or throughput or need an additional staining step. Other efforts focus on label‐free analysis of the calcification state and coccolith mass using polarization‐sensitive flow cytometry,^[^ ^22^, ^23^ ^]^ requiring expensive and specialized optics. Impedance flow cytometry has the advantage over optical flow cytometry, as it is easier and cheaper to integrate in compact Lab‐on‐a‐chip systems, but it lacks specificity (see perspective of ref. [24] for more details).

We therefore continue our earlier work on impedance flow cytometry, with novel components aimed to improve the sensitivity and throughput for assessing single‐cell PIC:POC ratios, and validate this using independent measurements of PIC:POC ratios of bulk cell material. Such a more sensitive single‐cell method may provide information on the variation in calcification state within a culture or population, in contrast to inorganic and organic carbon analysis in bulk. Impedance flow cytometry has been a widely studied and applied method^[^ ^25^, ^26^, ^27^, ^28^ ^]^ since its introduction two decades ago.^[^ ^29^ ^]^ Recent advances show the ability to 1) assess the viability of cryopreserved primary human PBMCs,^[^ ^30^ ^]^ 2) monitor the cell status of spheroids and microcarriers,^[^ ^31^ ^]^ 3) determine the deformability, cell size, and membrane properties of human neutrophils,^[^ ^32^ ^]^ and 4) use modified red blood cells as multimodal standard particles.^[^ ^33^ ^]^ Impedance flow cytometry provides cell features in a non‐invasive, label‐free, and high‐throughput (a few tens of cells per second for exploratory research^[^ ^32^ ^]^ up to several hundreds of cells per second for routine analysis^[^ ^25^ ^]^) manner. In general, the following features can be distinguished depending on the frequency with which we probe the cell: The cell size (<0.5 MHz), the cell membrane (1–10 MHz), and the cell's interior (>10 MHz).

Here, we hypothesize that we can evaluate the PIC:POC ratio of single coccolithophore cells by means of their phase response at high frequency. The cell interior is probed at high frequencies (>10 MHz), where the cell membrane impedance is negligible. This leads to a measurable electrical contrast between the conductive cell interior and non‐conductive (capacitive) calcium carbonate exoskeleton. Consequently, the phase response of calcified cells is expected to shift toward the phase response of non‐conductive (capacitive) polystyrene (PS) beads with increased calcification, because of their dielectric nature (Figure 1b). Likewise, calcification will increase the relative amount of PIC, thus shifting toward the phase response of PS beads, but also increasing the total electrical volume (Figure 1c). In contrast, photosynthesis (increasing POC) reduces the PIC:POC ratio and increases the electrical volume of the cell.

In this work we demonstrate a microfluidic impedance cytometer to measure the PIC:POC ratio of single coccolithophore cells at high‐throughput. We start by artificially modifying the calcite exoskeletons with acid to investigate the extremes and we assess the sensor's performance by comparing the results with inorganic and organic carbon analysis on bulk material (referred to as C analysis). Finally, we study the influence of changes in atmospheric CO_2_ levels and the population density on the PIC:POC ratio of coccolithophore cells, which is also verified by bulk C analysis.

## 2 Results and Discussion

### 2.1 Data Representation and Error Estimation

**Figure 2:**
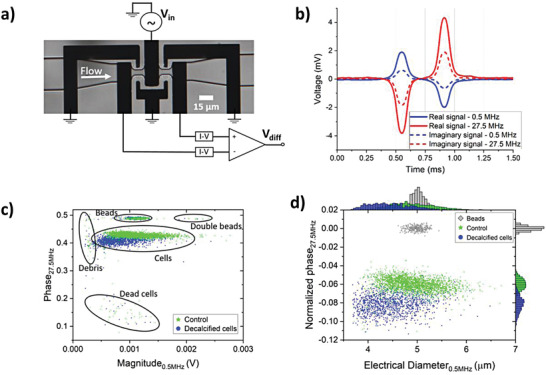
a) Overview of the differential impedance measurement setup showing the coplanar electrode pairs, which are shielded by the ground electrodes. The channel height is 10 µm. b) The measured real and imaginary signal response of a single coccolithophore cell recorded at 0.5 and 27.5 MHz. c) Phase and magnitude response of a control and acid treated population of cells (decalcified), both mixed with reference beads. Several subpopulations can be identified based on their electrical size and phase response. d) Processed data after normalization with respect to the reference beads and removal of debris, dead cells, and double beads. The electrical diameter is defined as the cubic root of the impedance magnitude (see Section 4.3 “Data acquisition and processing”).

The measurements were performed using a differential impedance measurement setup (**Figure** **2**a) as elaborately discussed in the material and methods section. We refer to Supporting Information for details on the processing of the complex impedance signal (Figure 2b), the phase and magnitude response (Figure 2c) and finally the differentiation and normalization of the measured particles (Figure 2d). Samples of coccolithophore cultures, grown exponentially under nutrient replete conditions, were directly introduced to the measurement system after spiking the sample with 5 µm PS reference beads. We observed multiple subsets of particles present in our samples (Figure 2c). We differentiate between beads, cells, small debris, and dead cells (Figures S2 and S3, Supporting Information). The size distribution of the reference beads (5.00 ± 0.14 µm for the beads in Figure 2d) is comparable to the standard deviation (±0.16 µm) given by the manufacturer, indicating that the electric field in the sensing area of the system is homogeneous (more details on the design in Section 4 “Device Design and Fabrication”). For the discussion of the next results, we only consider the group characterized as coccolithophore cells and the monodisperse reference beads.

### 2.2 Data Acquisition and Analysis

**Figure 3:**
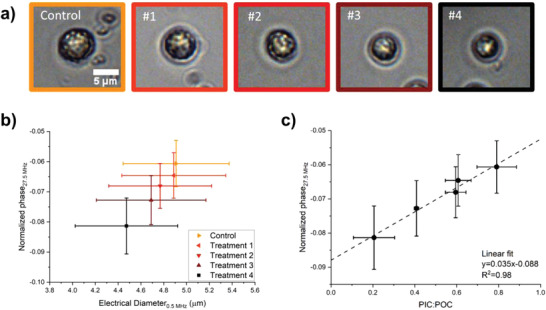
a) Optical representation of the mean calcification state for each acid treatment. The amount of added acid increases from left to right (#1 to #4). b) The mean normalized phase versus the mean electrical diameter of each treatment (*n* > 1000 for each treatment and indication of the standard deviation by the error bars). c) Calibration of the sensor: normalized phase versus the PIC:POC ratio of each treatment via bulk C analysis. The PIC:POC analysis was performed twice for each treatment and four times for the control group.

A control group of coccolithophore cells (no acid treatment) can be differentiated from a group of decalcified cells (dissolution of the exoskeleton by acid treatment), using the normalized phase (Figure 2d). A reduction in mean diameter was also observed, as expected, owing to the dissolution of the exoskeleton. Our findings strongly suggest that the phase is an independent measure of the PIC:POC ratio for two reasons. 1) The normalized phase of decalcified cells (100% POC) is approximately independent of their cell size (Figure S4, Supporting Information), thus their phase shift is independent of the absolute amount of organic content. 2) Single beads and double beads (corresponding to 100% PIC) have the same phase shift (Figure 2c), thus their phase shift is also independent of the absolute amount of inorganic content. Hence, suggesting that the phase is mainly dependent on the fraction of inorganic and organic content, as is made plausible by the experimental results of cells with modified fractions of inorganic content, which will be discussed in Section 2.3 (Figure 3 and Figure S1, Supporting Information).

We observed a large spread in normalized phase, which can be caused by the measurement system or the biological variation. We have investigated the source of this variation by quantifying the measurement error of individual particles (Figure S5a, Supporting Information) and compared this to error of a bulk measurement (Figure S5b, Supporting Information). From this comparison we can conclude that the biological variation is larger than our measurement error of single cells.

Another important aspect of the data interpretation is the robustness of the measurements. Changes in the measurement system (e.g., contaminations in the sensing region during testing or differences in electrode‐channel alignment between chips) can have a larger influence on the impedance response, than the subtle changes in the PIC:POC ratio we ultimately want to investigate (Figure S1, Supporting Information). Therefore, the magnitude and phase response of the PS beads were always checked to ensure a fair comparison between different sets of measurements, which we will get back to later.

### 2.3 Calcification Gradient and Calibration Measurement

To test the relationship between the normalized phase and the level of calcification, we created an artificial gradient in PIC:POC ratios through different acid treatments (**Figure** **3**a). The phase‐diameter plot shows a clear linear correlation (Figure 3b), as hypothesized (Figure 1c). Note that we here assume that changes in the cell diameter scale linearly with changes in the cell volume, which is true for small changes in diameter. The spread in size and phase can be explained by the large biological variation across single cells within a culture. Additionally, the acid treatment did not result in a fully homogeneous dissolution of the samples, as was observed by optical inspection of the strongest acid treatment. However, the mean dissolution of PIC is a proper representative of the mean change in PIC:POC ratio (>1000 cells measured per treatment).

We confirmed the relationship between the normalized phase and PIC:POC ratios with an independent method for quantifying the inorganic and organic carbon content in bulk coccolithophore culture (C analysis). Our results demonstrate a linear relation between the normalized phase and measured PIC:POC ratio (Figure 3c). More details on the bulk C analysis can be found in Figure S8, Supporting Information.

### 2.4 CO_2_ Experiment and Population Density

So far, we tested how artificial changes in the coccolithophore exoskeletons can be measured by normalized phase. The next step is to study changes in PIC:POC ratios as a result of natural perturbations in the carbon chemistry. To this end, we altered the carbon chemistry in culture media by aerating at a CO_2_ partial pressure (*p*CO_2_) of 400 and 1000 µatm (see Table S2, Supporting Information). We grew the coccolithophore strain *Emiliania huxleyi* 920/9 (CCAP, UK) under these different CO_2_ conditions, and assessed their growth over a period of 5–7 days. At the last day of each experiment, we analyzed the bulk C of the cultures. Although mean PIC:POC ratios measured with bulk C analysis tended to decrease in response to higher CO_2_ levels in three out of the four experiments, the changes were not significant (see Figures S6–S12, Supporting Information). Moreover, based on impedance measurements, the PIC:POC ratios remained largely unaltered in response to CO_2_ in all experiments. The unexpected lack of response to elevated *p*CO_2_ may have different causes. First, the response is different for every strain, some will be unaffected.^[^ ^34^ ^]^ Second, the physiological difference between the two treatments is relatively small and may become smaller after cells have grown as this causes a drift in the carbon chemistry (Table S2, Supporting Information). This is particularly evident in the experiments where cultures grew to higher cell densities (see also discussion below). Third, the technical error in our C analysis may possibly have overruled the differences between treatments, though this would not apply to the impedance measurements, in which we also did not observe changes in PIC:POC. Finally, other physiological changes, for instance the allocation of carbon to different macromolecules (carbohydrates, lipids), may possibly also alter the high frequency phase response and diminish the subtle difference between the CO_2_ treatments.

**Figure 4:**
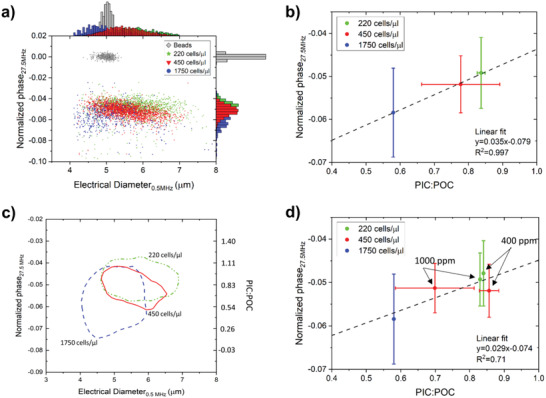
a) Normalized phase versus the electrical diameter for cultures at different cell densities. b) Comparison between the normalized phase and PIC:POC ratio versus the population density at 220 cells µL^−1^ (*n* = 2), 450 cells µL^−1^ (*n* = 6), and 1750 cells µL^−1^ (*n* = 1), ignoring the influence of the CO2 treatments. c) 75% population‐density contour plots for these cultures at different population density. The estimated PIC:POC ratio is shown on the right vertical axis and based on the calibration series in (b). d) The normalized phase versus the PIC:POC ratio for treatments of different population density and showing the different CO2 treatments. The error bars of the normalized phase are based on the spread (standard deviation) of all the single‐cell measurements per treatment (*n* > 450 per treatment). The standard deviation of the PIC:POC ratio is the result of multiple C analysis per treatment (see number of treatments at caption b).

**Figure 5:**
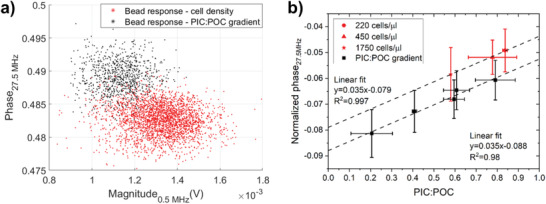
a) Raw impedance response of the reference beads for two sets of experiments (Figures 3 and 4) was different (i.e., changes in the measurement setup or chip). b) As a result there is shift in the normalized phase‐PIC:POC calibration, which stresses the importance of using reference beads. The error bars of the normalized phase are based on the spread (standard deviation) of all the single‐cell measurements per treatment. The standard deviation of the PIC:POC ratio is the result of multiple C analysis per treatment.

Perturbations in the carbonate chemistry can be prevented by limiting the population density. For example, in our experiments that reached a biomass of 170 cells µL^−1^, total dissolved inorganic carbon (DIC) was reduced by 13% (Table S2, Supporting Information), while in treatments with a higher biomass build‐up, this decrease was stronger. Comparing across the treatments thus not only involves variation in carbonate chemistry, and thereby PIC:POC ratios, but also differences because of variation in biomass build‐up. For impedance data, we can only compare those experiments where the beads showed similar responses (see discussion Figure 5). These experiments include the ones reaching 220 and 450 cells µL^−1^ in the exponential phase (at growth rates of *µ* = 0.83 d^−1^ and *µ* = 0.99 d^−1^, respectively), and the one of 1750 cells µL^−1^ in stationary phase (*µ* = 0.13 d^−1^). Across these experiments and CO_2_ treatments, we did observe a relationship between the measured PIC:POC ratio and normalized phase (**Figure** **4**). The highest PIC:POC ratios and larger cell sizes were observed under lower cell densities, where drift in the carbonate chemistry could be more constrained. Vice versa, with increasing cell densities, the carbonate chemistry drifted and led to lower PIC:POC ratios. We note, however, that there are many variables apart from the population density when comparing across experiments, for example, the time of analysis in the light:dark cycle was different, which may play a role in calcification as well.^[^ ^35^ ^]^ Yet, despite the lack of consistent responses toward changes in *p*CO_2_ and the variation across experiments, we did observe a clear correlation between the normalized phase and the PIC:POC ratio, showing the potential application for impedance measurements in detecting cellular changes in calcification.

The collection of all these single‐cell measurements gives unique information about the heterogeneity of each treatment (Figure 4c). A remarkable feature is the large variation in PIC:POC ratios for the cells from the experiment where biomass build‐up amounted to 1750 cells µL^−1^. From the 450 cells µL^−1^ data, we postulate that the larger the cell size, the smaller the relative size of the exoskeleton, based on the shape of the contour. These observations cannot be made with traditional C analysis, as these contain bulk culture material and thus represent an overall mean of many cells with various diameters and with various calcification states.

We can only compare different impedance measurements if the impedance response of the reference beads is equal, as mentioned before. The reference beads of the measurement series (in Figures 3 and 4) had a small shift in the mean magnitude and phase response compared to each other (**Figure** **5**a) and therefore we observe a shift in the calibration curve between the normalized phase and PIC:POC ratio as well (Figure 5b). Altogether, the similar trend in the normalized phase versus the PIC:POC ratio for both sets of experiments is a good indicator that our method is able to measure the average PIC:POC ratio via impedance.

### 2.5 Discussion

Impedance flow cytometry has several advantages compared to inorganic and organic carbon analysis in bulk: 1) It requires small amounts of sample (tens of µL versus tens of mL), 2) it is a real‐time and non‐invasive method, 3) it can be potentially integrated in other Lab‐on‐a‐chip systems, and 4) it gives information on the variation across cells within a culture or population. The major disadvantage is currently the poor robustness of our impedance sensor. At this point it was difficult to compare measurements done with different chips (i.e., slightly different alignment of the polydimethylsiloxane (PDMS) chip on the glass substrate with embedded electrodes), especially considering the relative small shift in phase we want to quantify. Therefore, the measurement system should be optimized, for example, by applying a different fabrication process to reduce the variation between chips. Additionally, we have studied only one species and strain (coccolithophore *E. huxleyi* 920/9), and it would be valuable to see if other algae phenotypes have a comparable response or need recalibration of the sensor. We also note that the PIC:POC approximation by the high frequency phase response ignores other physiological changes, for example, changes in the cell interior. In the future, recording an impedance spectrum and fitting an appropriate single cell model that captures all cell aspects might improve the determination of the PIC:POC ratio.

## 3 Conclusion

We have demonstrated an impedance‐based PIC:POC sensor for coccolithophore cells, enabling real‐time, non‐invasive, and high‐throughput assessment of the calcification state and size of calcifying algae. Artificial modification of the exoskeleton with several acid treatments resulted in a very strong linear fit (*R* ^2^ = 0.98) between the average normalized phase and PIC:POC ratio. The effect of different *p*CO_2_ treatments (400 and 1000 µatm) on the PIC:POC ratio of the coccolithophore *E. huxleyi* 920/9, however, was inconclusive. While PIC:POC ratios tended to decrease with elevated CO_2_ treatments, the variation was also high. We did find changes in the PIC:POC ratio as function of the population density. Cultures that grew to higher cell densities showed lower PIC:POC ratios, when testing across experiments and treatments, which was also recorded with our impedance‐based PIC:POC sensor. Additionally, this single‐cell method gives insight in the variation in size and PIC:POC within a culture of the same species or population and allows for real‐time tracking of a single‐cell or sample.

The development of this new quantification tool for small volumes of sample (and cells), paves the road for future analysis in high‐throughput systems while applying multi‐variable environmental stressors to predict the future marine carbon cycle.

## 4 Experimental Section

### Device Design and Fabrication

A large variety of electrode configurations have been presented over the years as recently reviewed by Zhu et al.^[^ ^36^ ^]^ A coplanar electrode configuration was designed and fabricated for differential impedance measurements (Figure 2a,b).^[^ ^37^ ^]^ These coplanar electrodes were easy to fabricate, but less sensitive, than planar (facing) electrodes. Nevertheless, the sensitivity of coplanar electrodes can be optimized with particular design choices:^[^ ^38^, ^39^, ^40^, ^41^ ^]^ A homogeneous electric field in the sensing region, a large electrode surface, a constriction in the channel, and shielding ground electrodes. The homogeneous electric field was realized by a small channel height (10 µm) with respect to the electrode separation (20 µm) and reduced the positional dependence of the particle in the sensing region. The large electrode surface increased the electrical double layer capacitance, thus reducing its influence at low frequency. The constriction channel (10 µm) reduced the total volume and therefore increased the sensitivity. Finally, the shielding ground electrodes reduced the stray capacitance and improved the signal‐to‐noise ratio. As a result the dielectric properties of single cells can be measured reliably and at high‐throughput in comparison to our earlier work.^[^ ^21^ ^]^

The device consisted of a PDMS chip, with channel structures, bonded on top of a glass chip with microelectrodes. A standard photolithography process was used to fabricate buried tantalum/platinum electrodes in a borosilicate glass wafer (SCHOTT MEMpax, 500 µm thickness) using a BHF wet etch, Ta/Pt sputtering and a lift‐off process. The 10 µm high SU‐8 mold for casting the PDMS chips (Sylgard 184, Dow Corning) was made using a well‐established lithography process. The bonding and the alignment of the glass‐PDMS chip was done with a custom made alignment tool.^[^ ^42^ ^]^

### Cell Cultivation and Preparation

Coccolithophore *E. huxleyi* 920/9 (CCAP, UK) was cultured at 16.5 ± 0.5 °C and at a photon flux density of 130 ± 15 µmol photons m^−2^ s^−1^ under a 16:8 light:dark cycle. Seawater from the north sea was filtered with a 0.22 µm syringe filter and enriched with macronutrients, vitamins, and trace metals according to F/2 medium.^[^ ^43^ ^]^ The maximum population density did not exceed 2 × 10^6^ cells mL^−1^. In the experiments testing for a CO_2_ effect, the population density did not exceed 6.0 × 10^5^ cells mL^−1^, to reduce the drawdown of DIC. Cell densities were measured by a counting chamber and the growth rate *µ* (d^−1^) was calculated according to

(1) $$\begin{array}{c} µ=\frac{\ln\left(\frac{C_{t}}{C_{0}}\right)}{t-t_{0}} \end{array}$$

with the population density *c* and the time *t* in days. Cell viability was monitored by checking the auto fluorescence of the chlorophyll. The number of dead cells was negligible under our test conditions. Moreover, cell viability after acid treatment was investigated by checking cell growth and regrowth of the exoskeleton. Both were observed within several hours after the acid treatment.

Artificial modification of the calcium carbonate exoskeleton was performed by acid treatment of the samples. First, strong acid (0.1 m HCl) was added to the sample (100 mL) to (partially) dissolve the exoskeleton. The pH was constantly monitored and restored to its initial condition (pH ≈ 8) after 2 min using a strong base (0.2 m NaOH). This procedure was followed to create four different acid treatments using 0.60, 0.75, 1.3, and 1.5 mL of strong acid (0.1 m HCl). A fifth sample was unmodified and used as control measurement. All samples originated from the same culture flask. The CO_2_ treatments (400 and 1000 µatm CO_2_ with synthetic air, Linde Gas) were prepared by continuously aeration of the culture medium and experimental systems (>30 mL min^−1^ in 100 mL culture). Impedance measurements were performed after five generation (five doubling times), requiring 4–6 days in total.

Samples for standard bulk C analysis^[^ ^44^ ^]^ were taken at the end of each experiment, and after the impedance measurements. To this end, 15–70 mL of culture (depending on the population density) was filtered over glass‐fiber filters (GF/F, 0.7 µm pore size, Whatman). The filters were stored at −20 °C and processed within 2 months after storage. Each glass‐fiber filter was cut in half, where one was used to determine the total particulate carbon (TPC) and the other for POC. Prior to the POC analysis, the PIC was dissolved by adding HCl (0.2 m, pure grade) to the filters. Parts of the filters (24%) were folded into a 5 × 8 mm tin cup for C analysis on a Flash 1112 EA Analyser (Interscience, Breda). PIC was calculated as the difference between the TPC and POC analysis.

PS reference beads of 5 µm (Sigma‐Aldrich) where mixed with each sample before the start of an impedance measurement. The mixture of cells and beads was pulled through the chip by applying a negative flow rate of −0.6 µL min^−1^ using a neMESYS syringe pump (Cetoni) and 100 µL syringe (Hamilton), resulting in a residence time of less than a millisecond in the sensing area (Figure 2b) and enabling a throughput of 2.5 to 20 cells s^−1^ depending on the population density.

### Data Acquisition and Processing

The complex impedance was recorded at 0.5 and 27.5 MHz simultaneously with a sampling rate of 115 kSa s^−1^ using a lock‐in amplifier (Zurich Instruments HF2LI) and transimpedance amplifier (Zurich Instruments HF2TA). The input signal was set to 2 *V*_peak‐to‐peak_ for both frequencies. The post processing of the impedance data was done in MATLAB (R2020a, MathWorks). A detailed description can be found in Supporting Information.

The impedance magnitude |*Z*| was recorded at low frequency (0.5 MHz), below the cell's β‐dispersion, to get information on the cell volume. The cell diameter scales with the cubic root of the impedance magnitude $k\sqrt[{3}]{|Z{|}_{0.5\text{MHz}}}$, where *k* is a calculated using the magnitude response of 5 µm PS beads. The phase was expressed at high frequency (27.5 MHz), above the cell's β‐dispersion, to get information on the cell's interior and thereby exploiting the electrical contrast between the conductive cell interior and the resistive calcite exoskeleton. The high frequency was set to 27.5 MHz arbitrary, well above the cell's β‐dispersion.

Cells, beads, debris and dead cells were identified from the raw data (Figure 2c). Typically particles smaller than 3.5–4.0 µm were classified as debris, this was verified by optical inspection (Figure S2, Supporting Information). Cells with a normalized phase response smaller than −0.12 (calibration series) and −0.10 (other measurements) are considered as dead cells (Figure S3, Supporting Information). Debris and dead cells were removed from the dataset for further processing of the results.

A contour plot was created using the 2D kernel density function in Origin (2021b, OriginLab Corporation). The contour was set to 75% of the cells and determined by integrating the cells within the area of the contour with respect to the total area. A statistical two sample t‐test was used to check whether the difference in normalized phase or PIC:POC ratio between the low and high CO_2_ treatment was significant (*p* < 0.05). The data was checked for normality using Shapiro‐Wilk (*p* < 0.05).

## Conflict of Interest

The authors declare no conflict of interest.

## Supporting information

Supporting Information

## Data Availability

The data that support the findings of this study are available from the corresponding author upon reasonable request.
