# Supplementary material for: Microfluidic Impedance Cytometry for Single‐Cell Particulate Inorganic Carbon:Particulate Organic Carbon Measurements of Calcifying Algae
Source: Glob Chall. 2022 Dec 7;7(3):2200151. doi: 10.1002/gch2.202200151 (PMC10000273; doi:10.1002/gch2.202200151)
Supplement: Supplementary file 1 — Supporting Information [file GCH2-7-2200151-s001.pdf]

Global Challenges
Open Access

Supporting Information

for *Global Challenges*, DOI: 10.1002/gch2.202200151

Microfluidic Impedance Cytometry for Single-Cell Particulate Inorganic Carbon:Particulate Organic Carbon Measurements of Calcifying Algae

*Douwe S.de Bruijn, Dedmer B. Van de Waal, Nico R. Helmsing, Wouter Olthuis, and Albertvan den Berg**

Supplementary Information

Microfluidic impedance cytometry for single-cell PIC:POC measurements of calcifying algae

Douwe S. de Bruijn[1,], Dedmer B. Van de Waal[2], Nico R. Helmsing[2], Wouter Olthuis[1], Albert van den Berg[1]*

[1]BIOS Lab-on-a-Chip group, MESA+ Institute for Nanotechnology, Max Planck – University of Twente Center for Complex Fluid Dynamics, University of Twente, The Netherlands

[2]Department of Aquatic Ecology, Netherlands Institute of Ecology (NIOO-KNAW), Droevendaalsesteeg 10, 6708 PB Wageningen, The Netherlands

[*]Correspondence: d.s.debruijn@utwente.nl

Device fabrication

A standard photolithography process was used to fabricate buried 10/135 nm tantalum/platinum electrodes in borosilicate glass wafers (SCHOTT MEMpax, 500 µm thickness). First a lift-off mask was created with photoresist to define the electrode pattern. Then a BHF wet etch was used to make 145 nm trenches, where after the tantalum and platinum layer were sputtered and later removed via lift-off. Lastly, the wafer was diced in separate chips.

Data processing

The differential impedance was measured with a lock-in amplifier (Zurich Instruments HF2LI), recording the real *X* and imaginary *Y* part of the signal. The baseline of the real and imaginary signal were removed by subtracting a seconder order polynomial fit and the signal noise was reduced by a moving average filter.

Next, particles/events were detected by the 'findpeak' algorithm of MATLAB. We observe an upward peak followed by a downward peak (or vice versa), owing to the nature of the differential measurement (Figure 2b). For each particle registration the value of *X* and *Y* was determined by the average of the first and the second peak. Then, we find the magnitude (*R)* and phase (ϕ) response as follows:

$$R = \sqrt{X^2 + Y^2}$$
$$\phi = \arctan\left(\frac{Y}{X}\right)$$

The signal response is not merely the result of the passing bead or cell, but it also influenced by the measurement system. E.g., cables will introduce a small time delay compared to the internal reference signal of the lock-in amplifier, which results in a significant phase shift of the baseline signal at high frequency. Therefore, the phase response is normalized with respect to the mean phase response of the reference beads (ϕ_{ref}) according to: $\phi_{\mathrm{normalized}} = \phi - \phi_{\mathrm{ref}}$. Note, that this normalization protocol differs from our earlier work ($\phi_{\mathrm{normalized}} = \frac{\phi}{\phi_{\mathrm{ref}}}$ [1]), resulting in a different phase range. The cell diameter was calculated as follows: $k\sqrt[3]{|Z|_{0.5\mathrm{MHz}}}$, where *k* is calculated using the magnitude response of 5 µm polystyrene beads.

Changes in the measurement system result in a different phase response of the reference beads as can be observed by the beads with the black color in Figure S1. The cells in the 'intermediate' (in red) and 'intermediate – shifted' (in black) run are from an identical treatment, unfortunately even after normalization we still observe a major shift in the phase response (Figure S1b), indicating that the phase shift of cells with respect to beads is nonlinear. In short, the bead response should be identical within measurements to make a fair comparison.

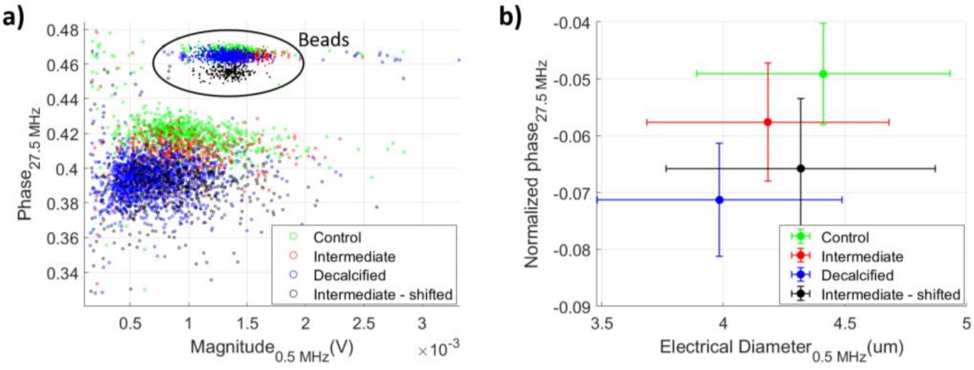

Figure S1: Robustness of the measurements. The measurement in red and black are the same treatment, but a change in the measurement system alters the bead response a) and results in a significant deviation, even after normalization b).

Finally, we discriminate between cells, beads, debris and dead cells (Figure 2c). Typically particles smaller than 3.5-4.0 µm are classified as debris and ignored, this was verified by optical inspection (e.g. Figure D.2). Cells with a normalized phase response smaller than -0.12 (calibration series) and -0.10 (other measurements) are also removed from the dataset as dead cells (Figure S3).

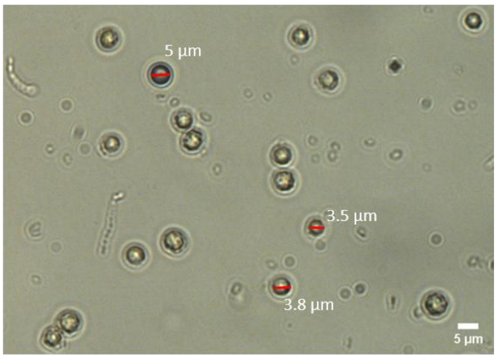

Figure S2: Image of decalcified cells and partially decalcified cells. The smallest decalcified cells are between 3.5-4.0 µm in diameter, smaller particles are labelled as debris and are excluded from further analysis. A 5 µm polystyrene bead is indicated as well.

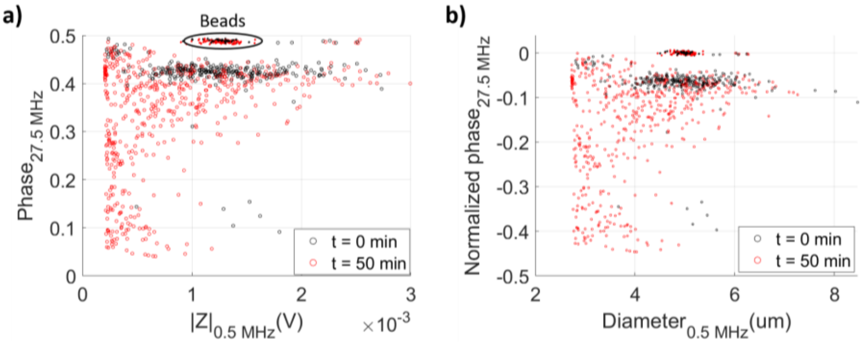

Figure S3: a) The raw phase and magnitude response before (t = 0 min) and after (t = 50 min) inducing cell death by adding surfactant (Tween-20) to the sample. A clear shift in phase and magnitude can be observed. b) Normalized phase and electrical diameter. All cells with a normalized phase < -0.1 are considered to be dead and are not taken into account for calculating the average phase response.

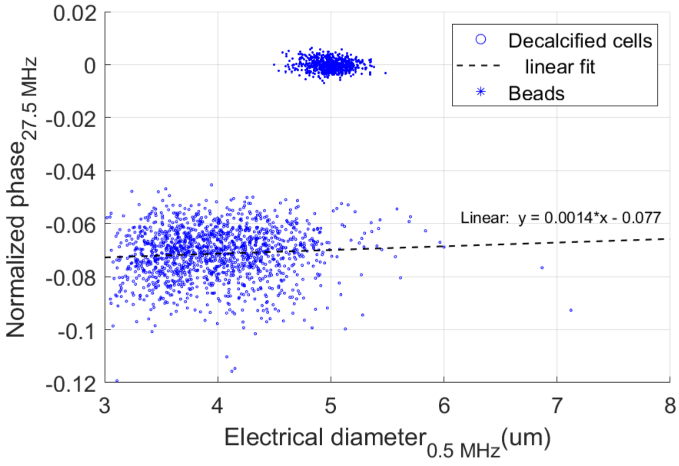

Figure S4: Phase-size relationship of decalcified cells. The phase at 27.5MHz is by approximation independent of the cell size.

Error estimation

We have quantified the measurement error of our system by moving an individual cell and two individual beads 10 to 20 times back and forth over the impedance sensor (Figure S5a). We can conclude that the biological variation is larger than our measurement error of single cells, based on the comparison of these single particles to a bulk measurement of hundreds of cells and beads (Figure S5b). We note that the variation in the single cell phase response is larger than of the single beads, possibly owing to different orientations of the possible non-uniformly shaped exoskeleton, which can be asymmetric.

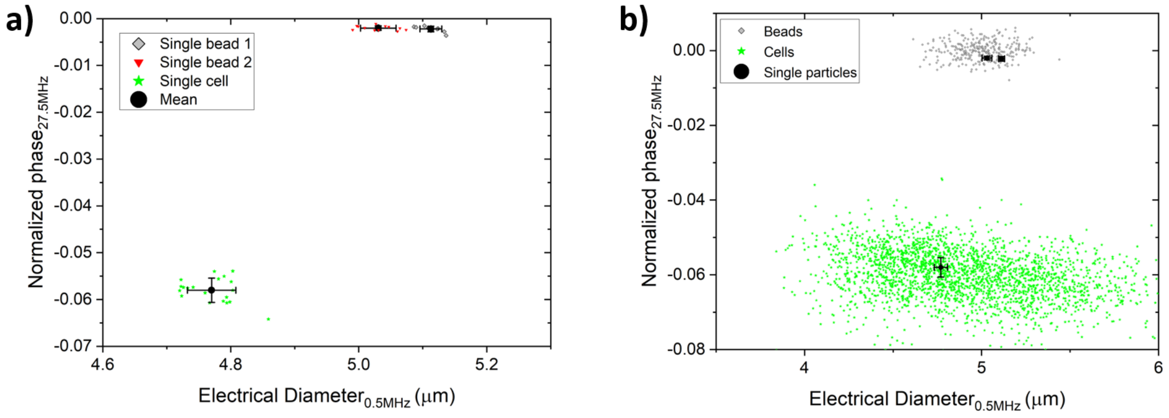

Figure S5: a) Error estimation of the normalized phase and electrical diameter of two single beads (bead 1: -0.0022±0.0006 and 5.11±0.02 µm, and bead 2: -0.0020±0.0005 and 5.03±0.03 µm) and a single cell (-0.0580±0.0026 and 4.77±0.04 µm). These single particles were measured 10 to 20 times by changing the flow direction. b) Comparison of the single particle measurement error and the bulk measurement of beads and cells. The error bars indicate the standard deviation of the individually measured particles.

CO_2 treatments and cell density additional data

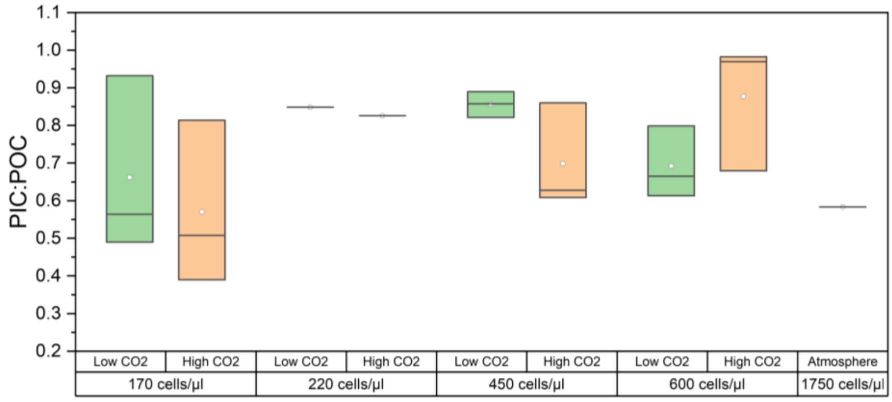

Figure S6: Boxplots of the PIC:POC ratio for different cell densities at low and high CO2 (400 and 1000 ppm, respectively). The difference between the low and high CO2 treatment is for none of the cell densities significantly different (t-test; P>0.05).

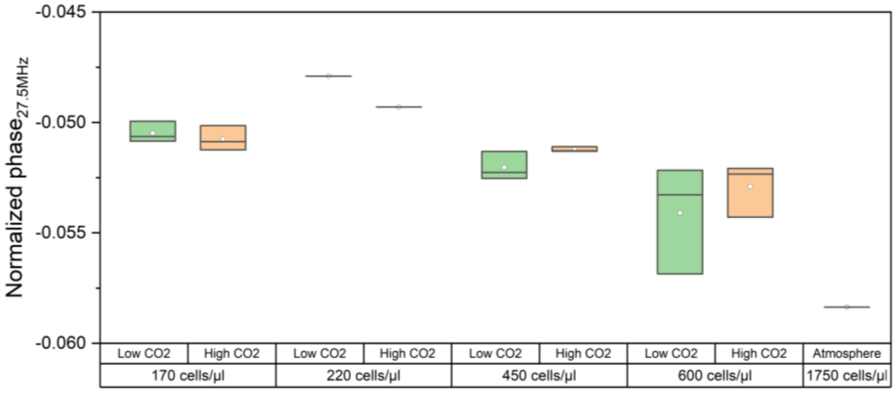

Figure S7: Boxplots of the normalized phase for different cell densities at low and high CO_2 (400 and 1000 ppm, respectively). The difference between the low and high CO_2 treatment is for none of the cell densities significantly different (t-test; P>0.05).

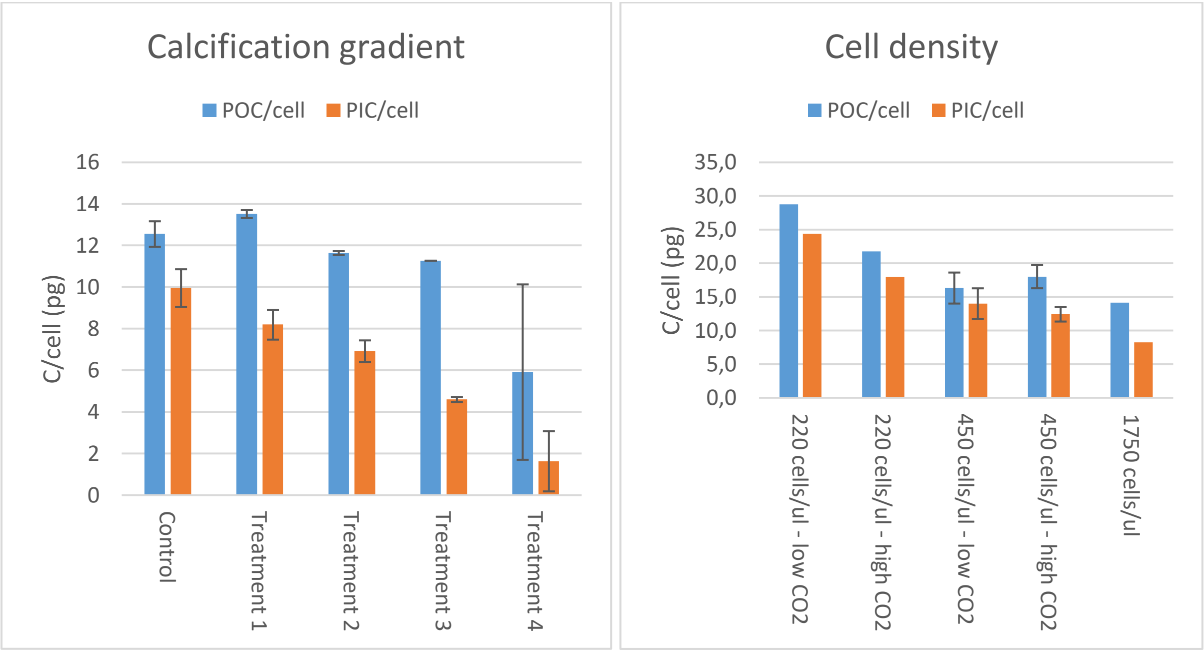

Figure S8: POC/cell and PIC/cell for the calcification gradient and cell density experiment.

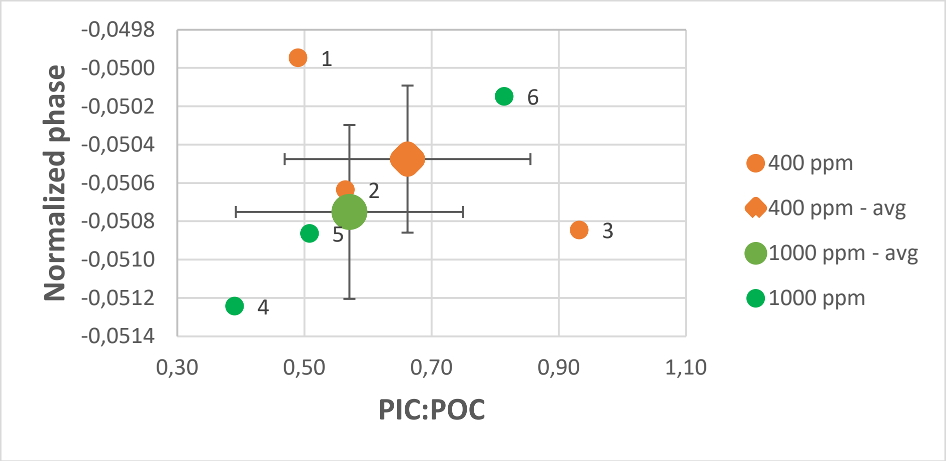

Figure S9: CO_2 experiment at cell density of ~170 cells/µl.

Table S1: Growth conditions for each sample in Figure S9.

Sample (#)	CO_2 concentration (ppm)	Cell density (cells/µl)	Growth rate (d^{-1})
1	400	170	0.77
2	400	160	0.72
3	400	160	0.71
4	1000	180	0.73
5	1000	160	0.64
6	1000	170	0.65

Table S2: Carbonate chemistry of the CO_2 experiment at ~170 cells/µl. CO2SYS output. Set of Constants: K1, K2 from Mehrbach et al., 1973 refit by Dickson and Millero, 1987. KHSO4: Dickson. pH Scale: NBS scale. [B]I Value: Uppstrom, 1974.

Sample (#)	CO_2 treatment (ppm)	Sampling moment	pH	DIC (mmol/L)	pCO_2 concentration (matm)	TA (mmol/L)
1	400	Start	8.04	1878	496	2038
		End	8.05	1613	415	1763
2	400	Start	8.05	1860	479	2024
		End	8.11	1578	351	1750
3	400	Start	8.05	1873	482	2037
		End	8.06	1583	398	1736
4	1000	Start	7.73	1948	1071	2005
		End	7.73	1765	971	1820
5	1000	Start	7.76	1858	953	1923
		End	7.95	1618	530	1734
6	1000	Start	7.74	1956	1051	2016
		End	7.75	1724	905	1784

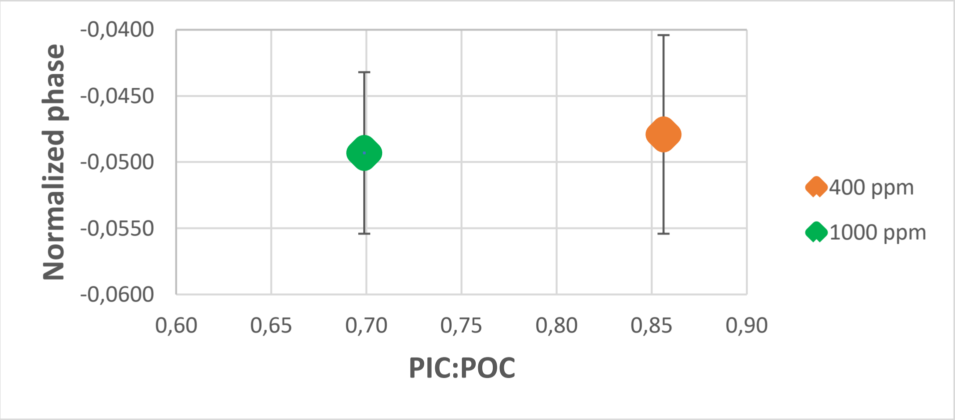

Figure S10: CO_2 experiment at cell density of ~220 cells/µl.

Table S3: Growth conditions for each sample in Figure S10.

Sample (#)	CO_2 concentration (ppm)	pH	Cell density (cells/µl)	Growth rate (d^{-1})
1	400	8.06	190	0.82
2	1000	7.78	250	0.83

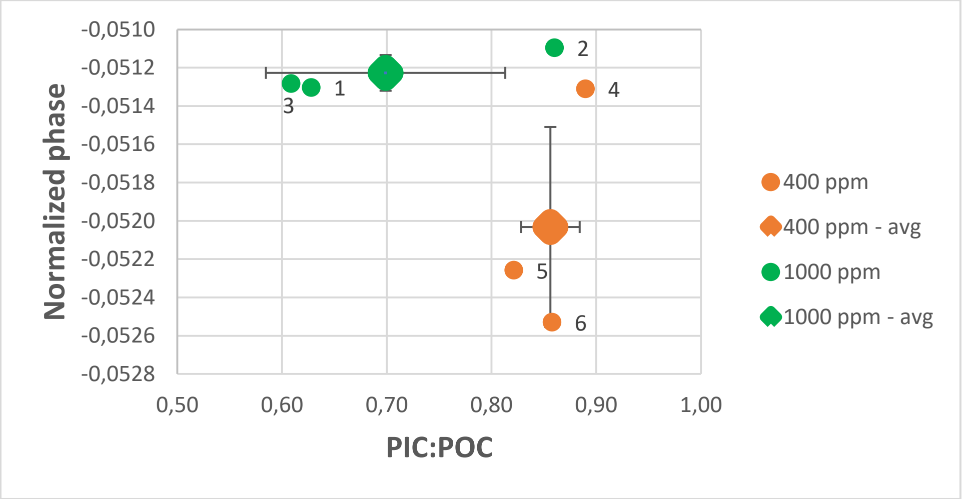

Figure S11: CO_2 experiment at cell density of ~450 cells/µl.

Table S4: Growth conditions for each sample in Figure S11.

Sample (#)	CO_2 concentration (ppm)	pH	Density (cells/µl)	Growth rate (d^{-1})
1	400	8.09	420	1.01
2	400	8.16	510	1.04
3	400	8.10	490	1.03
4	1000	7.79	430	0.98
5	1000	7.78	420	0.98
6	1000	7.82	380	0.96

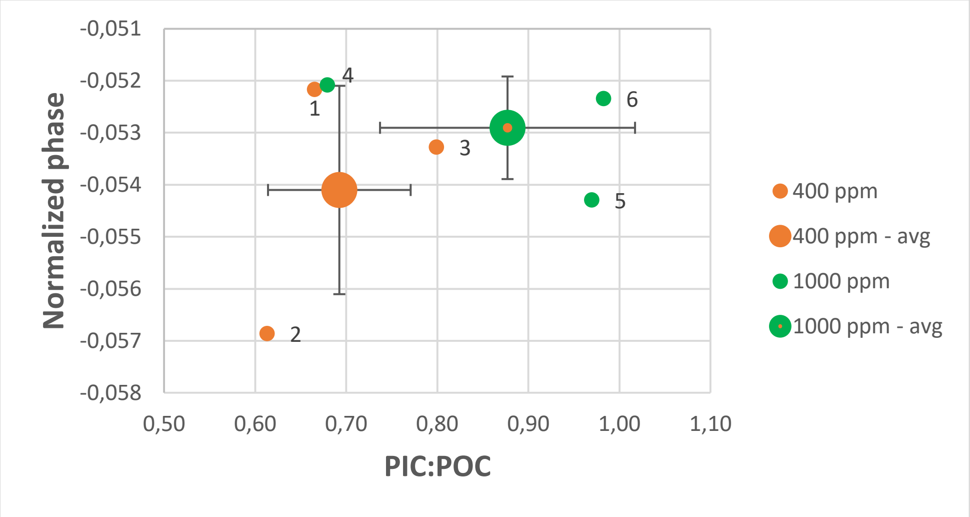

Figure S12: CO_2 experiment at cell density of ~600 cells/µl.

Table S5: Growth conditions for each sample in Figure S12.

Sample (#)	CO_2 concentration (ppm)	pH	Density (cells/µl)	Growth rate (d^{-1})
1	400	8.11	530	0.79
2	400	8.19	610	0.84
3	400	8.10	590	0.82
4	1000	7.91	630	0.84
5	1000	7.76	580	0.81
6	1000	7.80	590	0.82

Table S6: Growth conditions for the sample in stationary growth.

Sample (#)	CO_2 concentration (ppm)	pH	Cell density (cells/µl)	Growth rate (d^{-1})
1	~400 (direct contact atmosphere)	8.64	1750	0.13

References

[1] D. S. De Bruijn, P. M. Braak, D. B. Van De Waal, J. G. Bomer, W. Olthuis, and A. Van Den Berg, "Calcification State of Algae studied with Impedance Flow Cytometry," *25th Int. Conf. Miniaturized Syst. Chem. Life Sci. MicroTAS 2021*, no. October, pp. 559–560, 2021, Accessed: Nov. 15, 2022. [Online]. Available: https://research.utwente.nl/en/publications/calcification-state-of-algae-studied-with-impedance-flow-cytometr
